# Poor tuberculosis treatment outcomes in Southern Mozambique (2011–2012)

**DOI:** 10.1186/s12879-016-1534-y

**Published:** 2016-05-20

**Authors:** Alberto L. García-Basteiro, Durval Respeito, Orvalho J. Augusto, Elisa López-Varela, Charfudin Sacoor, Victor G. Sequera, Aina Casellas, Quique Bassat, Ivan Manhiça, Eusebio Macete, Frank Cobelens, Pedro L. Alonso

**Affiliations:** Centro de Investigação em Saude de Manhiça (CISM), Maputo, Mozambique; ISGlobal, Barcelona Ctr. Int. Health Res. (CRESIB), Hospital Clínic - Universitat de Barcelona, Barcelona, Spain; Amsterdam Institute for Global Health and Development, Academic Medical Centre, Amsterdam, The Netherlands; Ministry of Health, National Tuberculosis Program, Maputo, Mozambique

**Keywords:** Mortality, Tuberculosis, Manhiça, Death, Adverse outcome

## Abstract

**Background:**

In Mozambique, there is limited data regarding the monitoring of Tuberculosis (TB) treatment results and determinants of adverse outcomes under routine surveillance conditions. The objectives of this study were to evaluate treatment outcomes among TB patients, analyze factors associated with a fatal outcome and determine the proportion of deaths attributable to TB in the district of Manhiça, Southern Mozambique.

**Methods:**

This is a retrospective observational study based on TB patients diagnosed in the period 2011–2012. We used three different data sources: a) TB related variables collected by the National TB Control Program in the district of Manhiça for all TB cases starting treatment in the period 2011–2012. b) Population estimates for the district were obtained through the Mozambican National Statistics Institute. c) Deaths and other relevant demographic variables were collected from the Health and Demographic Surveillance System at Manhiça Health Research Center. WHO guidelines were used to define TB cases and treatment outcomes.

**Results:**

Of the 1957 cases starting TB treatment in the period 2011–2012, 294 patients (15.1 %) died during anti-tuberculous treatment. Ten per cent of patients defaulted treatment. The proportion of patients considered to have treatment failure was 1.1 %. HIV infection (OR 2.73; 95 % CI: 1.70–4.38), being female (OR: 1.39; 95 % CI: 1.31–1.91) and lack of laboratory confirmation (OR 1.51; 95 % CI: 1.10–2.08) were associated with dying during the course of TB treatment (p value <0.05). The contribution of TB to the overall death burden of the district for natural reasons was 6.5 % (95 % CI: 5.5–7.6), higher for males than for females (7.8 %; 95 % CI: 6.1–9.5 versus 5.4 %; 95 % CI: 4.1–6.8 respectively). The age group within which TB was responsible for the highest proportion of deaths was 30–34 among males and 20–24 among females (20 % of all deaths in both cases).

**Conclusion:**

This study shows a very high proportion of fatal outcomes among TB cases starting treatment. There is a high contribution of TB to the overall causes of mortality. These results call for action in order to improve TB (and TB/HIV) management and thus treatment outcomes of TB patients.

## Background

Tuberculosis (TB) remains an important public health concern and a leading cause of disease and death worldwide. According to the last global TB report it accounts for 9 million new cases and 1.5 million deaths per year [1]. Most of these deaths occur in Sub-Saharan Africa. Mozambique is one of the 22 high burden TB countries and, among the countries on that list, ranks third on high HIV co-infection rates with 56 % of TB patients being HIV positive [1]. The HIV epidemic, which has stricken the country deeply, hinders the millennium development goal of halting and beginning to reverse the incidence of TB by 2015 at a country level; a goal now achieved by most countries in the world.

Among the many challenges for TB control in Mozambique, ensuring proper TB diagnosis and treatment as well as implementing the one-stop model for TB and HIV care has been regarded as a national priority [2, 3]. A recent study conducted in Mozambique’s second largest city showed that patient and health system delay prior to TB treatment initiation is on average 150 days, which favors adverse outcomes during TB treatment [4]. In 2013, approximately 56,000 deaths were attributable to TB, although precise estimates are not available due the predictably low case detection rate and lack of information on cause of death, especially in people living with HIV [1]. Estimates at the national level speak of a treatment success rate (treatment complete and cured) of 87 % among the general population, which still does not meet the global targets for treatment success, set at 90 % by 2015 [5]. Worryingly, recent data from Manhiça district, in the south of the country, show an alarming mortality rate among adult TB cases co-infected with HIV [6]. The potential threat of increasing multi-drug resistance (MDR) numbers in the country, around 3.5 % among new cases according to the last national survey [7], could further jeopardize the achievement of the treatment success targets set in the strategic plan 2014–2018 by the National TB Control Program (NTP) [8].

Very little data are available in the country regarding the monitoring of treatment results and determinants of adverse outcomes under routine surveillance conditions. Knowledge on the burden of deaths attributable to TB among all causes of mortality is needed in order to set public health policy and prioritize interventions. The main objective of this study was to evaluate the treatment outcomes among TB patients and analyze factors associated with a fatal outcome. As a secondary objective, we estimated the proportion of deaths attributable to TB in a very high HIV- and TB-burden area in southern Mozambique.

## Methods

This is a retrospective observational study based on routine NTP data from TB patients diagnosed from January 2011 to December 2012 in the district of Manhiça, Southern Mozambique.

### Study area

The study was conducted at Manhiça Health Research Center (CISM from its acronym in Portuguese), located in the district of Manhiça, in Maputo province. CISM has a health and demographic surveillance system (HDSS) in the so-called ‘study area’, which at the time of this study was following approximately 92,000 individuals (covering around 53 % of the district population) living in 20,000 geo-positioned households. The system monitors important demographic events, such as births, deaths, migration movements and pregnancies, allowing for precise population estimates. An update of the health profile of the study area has been recently published elsewhere [9].

The prevalence of HIV infection in adults aged 18–47 was 39.9 % in 2010 [10] and the incidence of smear-positive TB among young adults living with HIV was 847 per 100,000 in 2011 [6]. A recent community incidence study in children under 3 revealed a minimum community incidence of 470 per 100,000 for this age group [11].

### Data sources

All TB related variables included in this study are part of the routine information collected by the NTP in the district of Manhiça for TB cases diagnosed in the period 2011–2012. Briefly, the NTP had, at that time, two offices in the district where TB patients could be registered and begin treatment: one at Manhiça’s District Hospital (HDM), and the other at the Rural Hospital of Xinavane, the second largest health facility in the district, which is located 53 km North of Manhiça village. The TB surveillance system is mostly passive, with non-systematic case finding strategies limited to TB contacts less than 5 years of age. Fatal outcome (death) is registered when a TB patient dies during TB treatment. Population estimates for the district were obtained based on the last official census (2007) through the Mozambican National Statistics Institute, and the estimated population for 2011 and 2012 using annual data from the HDSS at CISM. Demographic variables (sex, age, deaths, births, migrations) for the entire district were obtained extrapolating data from the HDSS to the entire district. From 2012 onwards, the HDSS recorded the type of death in those households who consented (accident, homicide, suicide, natural causes, disease, etcetera) in all deaths belonging to the study area (the proportion of TB attributable deaths among all deaths was therefore restricted to the year 2012). All data by CISM or NTP, although not freely available, can be accessed upon request.

### TB diagnosis, procedures and case definitions

Laboratory diagnosis of TB during 2011 was made on the basis of traditional microscopy using Ziehl-Neelsen staining. Culture was only done at diagnosis in patients previously treated for TB (former WHO Category 2 patients) or new TB cases who were acid-fast bacilli (AFB) smear positive at month 5 of follow up. Most AFB smears were processed in the Biosafety Level III laboratory at CISM, which was subjected to an external quality assurance program. Other samples from the north of the district were processed at the laboratory of the rural hospital of Xinavane. Pulmonary TB patients with confirmed TB are recommended to have sputum test at months 2 and 5 of follow up. WHO guidelines were used to define TB cases and treatment outcomes [12]. TB treatment is offered free of charge at central and peripheral health units. For first line treatment, fixed dose combinations in both the intensive and continuation phase are used in all country facilities. In the district of Manhiça, treatment is given on a weekly basis. A godfather or family relative is advised to oversee patient’s treatment. All children routinely receive Bacille Calmette-Guérin (BCG) vaccine at birth, with estimated coverage ranging from 86 to 94 % [13, 14].

### Data management and analysis

Data from the TB registries were entered into a Microsoft Access database (Microsoft Corporation, Redmond, WA, USA). Relevant variables included unique national TB identifier (NIT), age, name, TB diagnosis date, TB treatment initiation date, sex, type of TB (pulmonary or extrapulmonary), category of TB case (new/retreatment), HIV status, antiretroviral therapy (ART), outcome, and date of TB outcome. Data analysis was conducted using Stata, version 13 (Stata Corporation, College Station, TX, USA). TB profile by age and sex is depicted for the year 2011, since during 2012, a study involving active case finding in children under the age of three was being conducted in the area.

Univariate and multivariable logistic regression analyses were conducted to assess the effect of the different variables on our outcome variable “death during TB treatment”. All variables that showed at least some evidence of association (different Odds Ratio (OR) for the different categories and a Chi square p-value for statistical significance testing lower than 0.2 in the univariate analysis) were tested in the multivariable analysis. OR and 95 % confidence intervals and p-values were calculated. Pooled p-values were obtained for categorical variables through likelihood ratio tests. Those associations with p value less than 0.05 were regarded as statistically significant.

In order to build survival curves, the database containing TB surveillance data was linked with the Manhiça HDSS database. Common variables to both databases were used (name, age, gender and residence village). We used deterministic and probabilistic record linkage approaches through routines implemented in the RecordLinkage package on R 3.1.1 (R Development Core Team, Vienna, Austria). From this exercise, three lists were produced; the first depicted those patients with a perfect match; a second list with probable match and the third for possible match. The “probable” and “possible” match lists required further manual verification on the reason for discrepancy by personnel in the clinical and demographic departments. Kaplan Meier survival curves and log rank tests were then used to compare survival probabilities between different categories of the interest variable.

## Results

### Baseline characteristics and TB incidence rate

A total of 946 and 1011 TB cases started treatment in the district of Manhiça during 2011 and 2012, respectively. Fifty-six per cent of all cases diagnosed in the period 2011–2012 were male. The mean age at treatment initiation was 36.4 years (SD: 15.7) and 11.5 % were children below 15 years of age. The prevalence of HIV infection among those TB cases was 71.8 %. The proportion of HIV patients on ART at any time during treatment was 41.0 % (20.3 % and 62.2 % in 2011 and 2012 respectively, *p* <0.001). Eighty-three per cent of the cases had pulmonary TB and 10.1 % were previously treated for TB (Table 1). Among previously treated patients, 88.7 % were TB relapses or reinfections, 5.1 % were starting treatment after previous treatment failure and 6.2 % after treatment default. Thirty-three per cent of all TB cases were confirmed by smear microscopy (45.6 % and 2.2 % for pulmonary and extrapulmonary TB respectively).

**Table 1:** Univariate and multivariate analysis of factors associated with death during antituberculous treatment in the district of Manhiça (2011–2012)

	Total analysed		Outcome of treatment				uOR (95 % CI)	*p* value	aOR (95 % CI)	*p* value*
	N^#^	%	Death N	(%)	Alive N	(%)				
Year										
2011	946	48.3	157	16.6	789	83.4	*Ref*	0.063	*Ref*	0.87
2012	1011	51.7	137	13.6	871	86.4	0.79 (0.62–1.01)		1.03 (0.73–1.44)	
Age										
< 15	224	11.5	24	10.7	200	89.3	*Ref*	0.071	*Ref*	0.093
15–24	174	8.9	25	14.4	149	85.6	1.40 (0.77–2.54)		1.57 (0.49–5.01)	
25–34	625	32.1	91	14.6	534	85.4	1.42 (0.88–2.29)		1.40 (0.48–4.01)	
35–44	407	20.9	75	18.5	331	81.5	1.89 (1.15–3.09)		1.73 (0.58–5.14)	
45–54	258	13.2	34	13.2	223	86.8	1.27 (0.73–2.22)		1.56 (0.51–4.77)	
> 55	261	13.4	45	17.3	215	82.7	1.74 (1.03–2.97)		2.45 (0.80–7.50)	
Sex										
Female	856	43.9	118	13.8	738	86.2	*Ref*	0.15	*Ref*	0.041
Male	1096	56.2	176	16.1	917	83.9	1.20 (0.93–1.54)		1.39 (1.01–1.91)	
AFB smear at diagnosis										
Positive	750	51.7	89	11.9	658	88.1	*Ref*	0.005	*Ref*	0.009
Negative	701	48.3	120	17.1	581	82.9	1.52 (1.13–2.05)		1.54 (1.12–2.13)	
TB Category										
New Case	1746	89.9	260	14.9	1485	85.1	*Ref*	0.45		
Retreatment	195	10.1	33	16.9	162	83.1	1.16 (0.78–1.73)			
Type of TB										
Pulmonary	1630	83.4	237	14.6	1390	85.4	*Ref*	0.17	*Ref*	0.61
Extra-pulmonary	325	16.6	57	17.5	268	82.5	1.25 (0.91–1.71)		0.87 (0.52–1.47)	
HIV status										
HIV uninfected	540	28.2	45	8.4	494	91.7	*Ref*	<0.001	*Ref*	<0.001
HIV infected not on ART	811	42.4	164	20.2	647	79.8	2.78 (1.96–3.95)		3.50 (2.13–5.74)	
HIV infected on ART	563	29.4	70	12.5	491	87.5	1.56 (1.05–2.32)		1.83 (1.07–3.14)	

*uOR* unadjusted Odd Ratio, *aOR* Adjusted Odds Ratio, *ARV* On antiretroviral treatment, *AFB* acid fast bacilli

*p-value obtained through likelihood ratio test (LRT)

^#^In some variables (age, age, sex, AFB smear, TB category and TB type and HIV status) there is missing data, because these data were not available

**Fig. 1:**
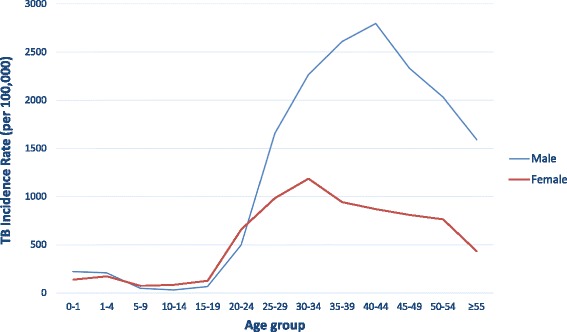
TB incidence rate in the district of Manhiça by sex and age group (year 2011)

Incidence rates for TB (including new cases and relapses) in the district of Manhiça were 540 and 571 cases per 100,000 population for 2011 and 2012, respectively. Incidence rates for bacteriologically confirmed TB were 212 and 218 per 100,000 population for 2011 and 2012. The highest incidence was observed among adults, being the highest among males aged 40–44 (2796/100,000) and females aged 30–34 years of age (1185/100,000) (Fig. 1).

### Treatment outcomes

Of all cases diagnosed with TB in the period 2011–2012, 294 patients (15.1 %) died during anti-tuberculous treatment. Treatment success (cured or treatment completed) occurred in 71.2 % of all patients (including new and retreatment cases). Ten per cent of patients defaulted treatment. The proportion of patients considered to have treatment failure was 1.1 % (only 703/1957 underwent smear at month 5). The proportion of treatment success among new cases was 72.4 %, with a success rate of 71.0 % among HIV positive patients, and 76.6 % among those with laboratory confirmed TB.

The univariate analysis showed that HIV-infected individuals had an odds of dying during TB treatment 2.3 times higher than among HIV uninfected TB patients. Among HIV positive patients, those who were not on ART were more likely to die during treatment (unadjusted uOR: 1.78 95 % CI 1.31–2.42). The multivariable logistic regression analysis showed that HIV infection (both patients on ART and not on ART, OR 1.83 95 % CI 1.07–3.14 and OR 5.50; 95 % CI: 2.13–5.74 respectively), being male (OR: 1.39; 95 % CI: 1.01–1.91) and lack of laboratory confirmation (OR 1.54; 95 % CI: 1.12–2.13) were associated with dying during the course of TB treatment (*p* value <0.05) (Table 1).

### Survival analysis

**Fig. 2:**
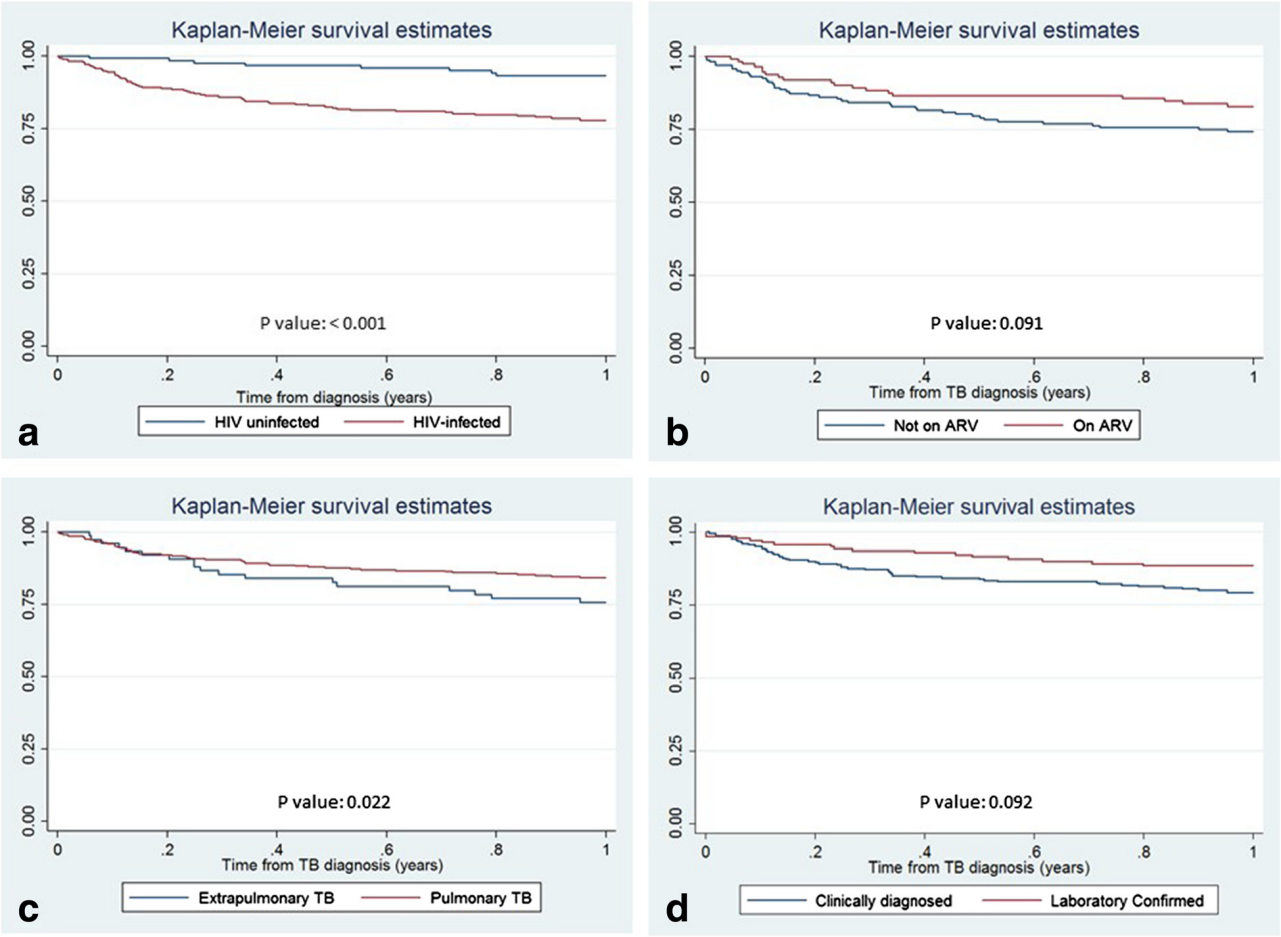
Kaplan-Meier survival estimates by **a) ** HIV status **b) ** ARV treatment **c) ** Type of TB **d) ** Laboratory confirmation (year 2011–2012)

The total number of TB patients diagnosed in 2011–2012 who were linked to the demographic surveillance system database was 395 (38.1 % of estimated TB cases belonging to the study area). Kaplan–Meier estimates for the probability of surviving 1 year after treatment initiation, by HIV status, type of diagnosis, type of TB and being on ART are presented in Fig. 2. The cumulative probability of survival 6 months after TB treatment initiation had begun was 0.87 and 1 year after treatment was 0.83. Patients with TB/HIV co-infection were less likely to be alive at the end of first year after treatment initiation compared to TB HIV uninfected patients (survival probability: 0.78 and 0.93 respectively, *p* value <0.001). Likewise, 76 % of the extrapulmonary TB patients remained alive 1 year after treatment initiation compared to 85 % of pulmonary TB cases (*p* value <0.022).

### Burden of TB associated mortality

During the year 2012, there were an estimated 2120 deaths in the district of Manhiça. Excluding violent deaths (road and household accidents, homicides and suicides), which accounted for 4.7 % of the total deaths, there were 2019 estimated deaths (951 and 1068 among males and females) attributable to disease or natural causes. The contribution of diagnosed TB to the overall death burden of the district for natural reasons was 6.5 % (132/2019) (95 % CI: 5.5–7.6) (Table 2), higher for males than for females (7.8 %; 95 % CI: 6.1–9.5 versus 5.4 %; 95 % CI: 4.1–6.8 respectively), although not statistically significant. The age group within which TB was responsible for the highest proportion of deaths was 30–34 among males and 20–24 among females (20 % of all deaths in both cases).

**Table 2:** Proportion of deaths attributable to Tuberculosis in the district of Manhiça. Year 2012

	All population			Males			Females		
Age group	Deaths District^a^	TB related deaths	% Deaths (95 % CI) associated to TB	Deaths District^a^	TB related deaths	% Deaths (95 % CI) associated to TB	Deaths District^a^	TB related deaths	% Deaths (95 % CI) associated to TB
0–1	232	0	0.0 (0.0–0.0)	112	0	0.0 (0.0–0.0)	120	0	0.0 (0.0–0.0)
1–4	242	5	2.1 (0.3–3.9)	112	2	1.8 (0.0–4.2)	130	3	2.3 (0.0–4.9)
5–9	56	1	1.8 (0.0–5.3)	39	0	0.0 (0.0–0.0)	17	1	5.74 (0.0–17.1)
10–14	35	1	2.9 (0.0–8.4)	23	0	0.0 (0.0–0.0)	12	1	8.6 (0.0–24.0)
15–19	40	0	0.0 (0.0–0.0)	19	0	0.0 (0.0–0.0)	21	0	0.0 (0.0–0.0)
20–24	60	11	18.3 (8.5–28.1)	19	3	15.8 (0.0–32.2)	41	8	19.7 (7.4–31.6)
25–29	104	15	14.4 (7.7–21.2)	50	9	17.9 (7.4–28.6)	54	6	11.1 (2.7–19.5)
30–34	168	30	17.9 (12.1–23.6)	95	19	20.0 (12.0–28.0)	74	11	14.9 (6.8–23.0)
35–39	162	21	13.0 (7.8–18.1)	75	12	15.9 (7.7–24.3)	87	9	10.3 (3.9–16.7)
40–44	110	15	13.6 (7.2–20.0)	66	9	13.7 (5.4–21.9)	44	6	13.5 (3.5–23.8)
45–49	108	5	4.6 (0.7–8.6)	56	3	5.3 (0.0–11.3)	52	2	3.8 (0.0–9.1)
50–54	107	9	8.5 (3.2–13.8)	37	7	19.0 (6.3–31.5)	70	2	2.9 (0.0–6.8)
≥55	594	19	3.2 (1.8–4.6)	248	10	4.0 (1.6–6.5)	346	9	2.6 (0.9–4.3)
Total	2019	132	6.5 (5.5–7.6)	951	74	7.8 (6.1–9.5)	1068	58	5.4 (4.1–6.8)

^a^Excluding violent or accidental deaths (car or at home), suicides and homicides

## Discussion

This study is one of the few operational research studies assessing TB treatment outcomes and mortality burden attributable to TB in Mozambique. We found an extremely high fatality rate among patients on TB treatment (15.1 %), much higher than recent figures from neighboring sub-Saharan African countries [15–17]. Moreover, the general contribution of TB to the overall causes of mortality in our district is very high (6.5 %), skyrocketing up to one out of every five deaths among young adult females and males in their mid-thirties.

Tuberculosis was an important cause of death in both 2011 and 2012, especially among middle age groups. Although the analysis approach does not allow comparison among other diseases, it is very likely that TB ranks among the leading causes of death. In South Africa, during 2013, tuberculosis was the leading cause of death, accounting for 8.8 % of all deaths, and of 9.8 % of all deaths for natural causes [18]. As in South Africa, our study shows that the proportion of deaths due to tuberculosis among all deaths by natural causes was higher for males (7.8 %) compared to females (5.4 %). Due to the difficulty of ascertaining the actual cause of deaths of TB patients, we have defined death due to tuberculosis as a death occurring during TB treatment. This certainly overestimates the true TB attributable deaths among patients on treatment, since dying during TB treatment does not equal dying of TB. However, since our analysis is based on deaths among notified TB cases only and WHO estimated a case detection rate of 34 % in the country during 2012, it is very likely that it underestimated the true TB contribution to the death burden. To accurately ascertain cause of death by TB, the only acceptable method would be the postmortem evaluation of those cases [19, 20].

The treatment success rate (which includes those cured or who completed treatment) in the district of Manhiça for the period 2011–2012 was 71.2 %, and 76.6 % among those laboratory confirmed TB patients. More than 50 % of the cases who did not achieve treatment success died (defaulting, treatment failure and transfer out cases account for the other cases). These results are far below the 87 % treatment success target set by the Stop TB partnership for the year 2015 and even further away from the new target (90 %) of the Global Plan to Stop TB 2011–2015 [5]. Although at a country level Mozambique reported treatment success rates of 87 % among new cases [1] for the year 2013 (no data are available for 2012), the lower figures obtained in the district of Manhiça might have different explanations. HIV prevalence among TB cases in the district of Manhiça has been reported to be higher than national level estimates [6]. Our study and the literature repeatedly report a higher proportion of poorer outcomes among HIV-infected individuals [15, 21–24]. Among HIV patients, the proportion of patients on ART (at any time during treatment) was very low (41.0 %). This contributes to the overall poor outcomes [17, 25]. The reason behind better treatment outcomes in 2012 than 2011 is driven primarily by a higher proportion of patients on ART (the difference disappears after adjusting for HIV infection and ART status in the multivariable regression model). Interestingly, there is substantial 4 % additional mortality from 6 month to 1 year after treatment initiation (when most patients have already finished treatment). This could be attributable to clinical conditions related to HIV/AIDS, although cannot explain all the additional mortality after 6 month treatment, since some of those deaths occurring 6 months after treatment initiation were in HIV negative individuals. High defaulting treatment rate (10 %) and poor adherence to TB treatment (which would translate into longer treatment periods), but not necessarily defaulting, could also be another factor for low success rate, although the length of TB treatment was not assessed in our study.

Those TB cases who were not laboratory confirmed with smear microscopy had a higher odds of dying during treatment than those who were just clinically diagnosed (Table 1 and Fig. 2b). This might have two different explanations. Some may have been misdiagnosed with TB, thus antituberculous therapy was useless and could have delayed or prevented access to the correct treatment for their condition. Another explanation would be that HIV positive individuals have higher immunosuppression (lower CD4 counts) and are often paucibacillary, thus less likely to have a positive smear. This may have led to diagnostic delays, contributing to the poorer outcomes [26]. Although several studies have shown that re-treatment cases are more likely to have a poor outcome [26], mostly as a consequence of higher rates of defaulting and higher MDR rates, we could not show this association in our study. A potential explanation for this is that information on previous TB treatment might have not been properly recorded (patients’ recall bias or lack of adequate history taking). Only 10 % of patients were classified as retreatment, while, for example, in Cape Town, South Africa, retreatment cases consistently account for 30 % of notified TB cases [27, 28].

Although TB/HIV patients not on antiretroviral treatment were significantly more likely to have a fatal outcome than those taking ART (20.3 % vs 12.5 %, *p* < 0.001), we expected to find a greater difference [29]. Kaplan Meier survival analysis, although conducted on a smaller sample, did not show statistical differences between both groups. Besides the small numbers, this might be due to the fact that patients who received ART were also those at highest risk of dying (because they had low CD4 counts or other opportunistic infections). An alternative explanation could be the poor reporting on ART status before February 2012, when the HIV/TB one-stop model was implemented in the district (HIV treatment during TB treatment being provided and monitored by the NTP officers). In fact the proportion of HIV infected TB patients on ART increased sharply from 2011 to 2012 (20 % to 62 % respectively). Thus, some patients might have started HIV treatment during TB treatment also in 2011, but this information may have been poorly or not registered on the TB registry books. In fact, a recent study in Manica Province (Central Mozambique), reported that only 73 % of data on ART use in the TB registry were correct [30].

This study had several limitations. The linking of the NTP registry book with the HDSS dataset could only be done to a subset of patients belonging to the study area, limiting the sample size for the survival analysis. The reasons for this are the different names used by patients, handwriting errors on the matching variables and the impossibility of verification on those without a perfect matching. That said, we believe there are no sources of selection bias in the sample used for this analysis. Second, we could not assess the impact of other important variables/comorbidities on mortality, such us malnutrition, diabetes, occupation or immunosuppression (CD4 counts), which would provide further characterization of TB mortality factors. Third, the aforementioned potential misreporting of use of ARTs or TB type (new case or retreatment) calls for cautious interpretation of the magnitude of association of mortality and these two variables. Fourth, during the study period, culture was not routinely performed, thus some false positive smear results, due to non tuberculous mycobacteria (NTMs) could have occurred. However, this might have had limited impact on treatment outcomes, since the proportion of NTMs among positive smears from adult population is below 2 % (unpublished data). Lastly, due to the lack of reliable information on dates of death and defaulting and very few deaths on the subsample that could be linked to HDSS, Cox regression, a preferred analysis strategy for analysis factors associated to mortality, was not performed.

## Conclusions

This study contributes to the limited surveillance-based analyses on TB programmatic indicators at district level in a high TB/HIV burden country. Our results show that fatal outcomes among TB cases starting treatment are very high. There is a need for improving the proportion of TB/HIV cases being linked to HIV care. The study also showed a high contribution of TB to the overall causes of mortality suggesting that TB remains one of the major public health problems in the country. These results call for renewed action in order to improve management and thus treatment outcomes of TB patients.

## Abbreviations

AFB: acid fast bacilli
AIDS: Acquired Immunodeficiency Syndrome
ART: antiretroviral therapy
BCG: Bacille Calmette-Guérin
CISM: Manhiça Health Research Center
HDSS: Health and Demographic Surveillance System
HDM: Manhiça District Hospital
HIV: Human Immunodeficiency Virus
MDR: multi-drug resistance
NIT: TB identifier
NTP: National Tuberculosis Program
OR: odds ratio
TB: tuberculosis
WHO: World Health Organization

## References

[1] World Health Organization (2014). Global Tuberculosis Report 2014.

[2] García-Basteiro AL, López-Varela E, Manhiça I, Macete E, Alonso PL (2014). Mozambique faces challenges in the fight against tuberculosis. Lancet.

[3] Plano Estrategico Nacional de Controlo da Tuberculose em Moçambique para o periodo 2008–2012. http://www.who.int/countries/moz/publications/tb_national_strategy.pdf Accessed 15 May 2016.

[4] Saifodine A, Gudo PS, Sidat M, Black J (2013). Patient and health system delay among patients with pulmonary tuberculosis in Beira city, Mozambique. BMC Public Health.

[5] The Global Plan to Stop TB (2011–2015). http://stoptb.org/assets/documents/global/plan/TB_GlobalPlanToStopTB2011-2015.pdf Accessed 13 May 2016.

[6] Garcia-Basteiro A, Lopez-Varela E, Respeito D, Gonzalez R, Naniche D, Manhiça I, Macete E, Cobelens F, Alonso P (2015). High Tuberculosis Burden among People living with HIV in Southern Mozambique. Eur Respir J.

[7] Samo Gudo P, Cuna Z, Coelho E, Maungate S, Borroni E, Miotto P, Ahmadova S, Brouwer M, Migliori GB, Zignol M, Cirillo DM (2011). Is multidrug-resistant tuberculosis on the rise in Mozambique? Results of a national drug resistance survey. Eur Respir J Off J Eur Soc Clin Respir Physiol.

[8] Ministry of Health of Mozambique. Plano Estrategico e Operacional (2014-2018). Maputo, Mozambique; 2014.

[9] Sacoor C, Nhacolo A, Nhalungo D, Aponte JJ, Bassat Q, Augusto O, Mandomando I, Sacarlal J, Lauchande N, Sigaúque B, Alonso P, Macete E (2013). Profile: Manhica Health Research Centre (Manhica HDSS). Int J Epidemiol.

[10] González R, Munguambe K, Aponte J, Bavo C, Nhalungo D, Macete E, Alonso P, Menéndez C, Naniche D (2012). High HIV prevalence in a southern semi-rural area of Mozambique: a community-based survey. HIV Med.

[11] Lopez-Varela E, Augusto O, Gondo K, Garcia-Basteiro A, Fraile O, Ira T, Ribó-Aristizabal J, Bulo H, Muñoz J, Aponte J, Macete E, Sacarlal J, Alonso P. Incidence of Tuberculosis among young children in rural Mozambique. PIJD. 2015. In press. 10.1097/INF.0000000000000710 26069945

[12] World Health Organization. Definitions and Reporting Framework for Tuberculosis – 2013 Revision. WHO/HTM/TB/2013.2; 2013.

[13] Consonni D, Montenegro Agorostos Karagianis MM, Bufardeci G (2013). Immunisation with BCG in the Maringue District, Sofala Province, Mozambique. Tuberc Res Treat.

[14] World Health Organization. WHO vaccine-preventable diseases: monitoring system. Mozambique: Global Summary; 2013. http://apps.who.int/immunization_monitoring/globalsummary/countries?countrycriteria%5Bcountry%5D%5B%5D=MOZ&commit=OK Accessed 13 May 2016.

[15] Field N, Lim MS, Murray J, Dowdeswell RJ, Glynn JR, Sonnenberg P (2014). Timing, rates, and causes of death in a large South African tuberculosis programme. BMC Infect Dis.

[16] Hamusse SD, Demissie M, Dejene T, Lindtjom B (2014). Fifteen-year trend in treatment outcomes among patients with pulmonary smear-positive tuberculosis and its determinants in Arsi Zone, Central Ethiopia. Gobal Heal Action.

[17] Tweya H, Feldacker C, Phiri S, Ben-Smith A, Fenner L, et al. Comparison of Treatment Outcomes of New Smear-Positive Pulmonary Tuberculosis Patients by HIV and Antiretroviral Status in a TB/HIV Clinic, Malawi. PLoS ONE. 2013;8(2):e56248. doi: 10.1371/journal.pone.0056248 10.1371/journal.pone.0056248 PMC3574145 23457534

[18] Mortality and causes of death in South Africa, 2013: Findings from death notification / Statistics South Africa. Pretoria: Statistics South Africa; 2014. http://beta2.statssa.gov.za/publications/P03093/P030932013.pdf Accessed 13 May 2016.

[19] Garcia-Basteiro AL, Mamudo I, Carrilho C, Ussene E, Castillo P, Chitsungo D, Rodríguez C, Lovane L, Vergara A, Lorenzoni C, Ordi J, Menéndez C, Bassat Q, Martínez MJ. The role of Xpert MTB/RIF in diagnosing pulmonary tuberculosis in post-mortem tissues. Sci Rep. 2016;6:20703. 10.1038/srep20703 PMC4748254 26860394

[20] Bassat Q, Ordi J, Vila J, Ismail MR, Carrilho C, Lacerda M, Munguambe K, Odhiambo F, Lell B, Sow S, Bhutta ZA, Rabinovich NR, Alonso PL, Menéndez C (2013). Development of a post-mortem procedure to reduce the uncertainty regarding causes of death in developing countries. Lancet Glob Heal.

[21] Ade S, Harries AD, Trébucq A, Ade G, Agodokpessi G, Adjonou C, Azon S, Anagonou S (2014). National profile and treatment outcomes of patients with extrapulmonary tuberculosis in Bénin. PLoS One.

[22] Kirenga BJ, Levin J, Ayakaka I, Worodria W, Reilly N, Mumbowa F, Nabanjja H, Nyakoojo G, Fennelly K, Nakubulwa S, Joloba M, Okwera A, Eisenach KD, McNerney R, Elliott AM, Mugerwa RD, Smith PG, Ellner JJ, Jones-López EC (2014). Treatment outcomes of new tuberculosis patients hospitalized in Kampala, Uganda: A prospective cohort study. PLoS One.

[23] Shuldiner J, Leventhal A, Chemtob D, Mor Z (2014). Mortality of tuberculosis patients during treatment in israel, 2000–2010. Int J Tuberc Lung Dis.

[24] Sanchez M, Bartholomay P, Arakaki-Sanchez D, Enarson D, Bissell K, Barreira D, Harries A, Kritski A (2012). Outcomes of TB treatment by HIV status in national recording systems in Brazil, 2003–2008. PLoS One.

[25] Addis Alene K, Nega A, Wasie Taye B (2013). Incidence and predictors of tuberculosis among adult people living with human immunodeficiency virus at the University of Gondar Referral Hospital, Northwest Ethiopia. BMC Infect Dis.

[26] Munoz-Sellart M, Cuevas LE, Tumato M, Merid Y, Yassin MA (2010). Factors associated with poor tuberculosis treatment outcome in the Southern Region of Ethiopia. Int J Tuberc Lung Dis.

[27] Cape Town TB Control - Progress Report 1997–2003. http://www.hst.org.za/publications/cape-town-tb-control-progress-report-1997–2003 Accessed 13 May 2016.

[28] Middelkoop K, Bekker L-G, Shashkina E, Kreiswirth B, Wood R (2012). Retreatment tuberculosis in a South African community: the role of re-infection, HIV and antiretroviral treatment. Int J Tuberc Lung Dis.

[29] Karim SSA, Naidoo K, Grobler A, Padayatchi N, Baxter C, Gray A, Gengiah T, Nair G, Bamber S, Singh A, Khan M, Pienaar J, El-sadr W, Friedland G, Karim QA (2010). Timing of Initiation of Antiretroviral Drugs during Tuberculosis Therapy. N Engl J Med.

[30] Brouwer M, Gudo PS, Simbe CM, Perdigão P, van Leth F (2013). Are routine tuberculosis programme data suitable to report on antiretroviral therapy use of HIV-infected tuberculosis patients?. BMC Res Notes.

