# Erratum to: Poor tuberculosis treatment outcomes in Southern Mozambique (2011-2012)

**DOI:** 10.1186/s12879-016-1948-6

**Published:** 2016-10-25

**Authors:** Alberto L. García-Basteiro, Durval Respeito, Orvalho J. Augusto, Elisa López-Varela, Charfudin Sacoor, Victor G. Sequera, Aina Casellas, Quique Bassat, Ivan Manhiça, Eusebio Macete, Frank Cobelens, Pedro L. Alonso

**Affiliations:** 1 Centro de Investigação em Saude de Manhiça (CISM), Maputo, Mozambique; 2 ISGlobal, Barcelona Ctr. Int. Health Res. (CRESIB), Hospital Clínic - Universitat de Barcelona, Barcelona, Spain; 3 Amsterdam Institute for Global Health and Development, Academic Medical Centre, Amsterdam, The Netherlands; 4 Ministry of Health, National Tuberculosis Program, Maputo, Mozambique

## Erratum

Following publication of the original article [1], the authors became aware that a sentence in the abstract was not corrected from a previous version of the manuscript.

Instead of: “HIV infection (OR 2.73; 95 % CI: 1.70-4.38), being female (OR: 1.39; 95 % CI 1.31-1.91) and lack of laboratory confirmation (OR: 1.51; 95 % CI 1.10-2.08) were associated with dying during the course of TB treatment (*p* value <0.05)”.

It should read: “HIV infection (OR 2.73; 95 % CI: 1.70-4.38), being male (OR: 1.39; 95 % CI 1.01-1.91) and lack of laboratory confirmation (OR: 1.54; 95 % CI 1.12-2.13) were associated with dying during the course of TB treatment (*p* value <0.05)”.

Please note that none of the conclusions of this manuscript are altered as a result of this modification.
